# CLINICAL APPROACHES TO SUPPORT MALE DEMENTIA FAMILY CAREGIVERS

**DOI:** 10.1093/geroni/igae098.0123

**Published:** 2024-12-31

**Authors:** Carolyn Clevenger

**Affiliations:** Emory University, Atlanta, Georgia, United States

## Abstract

Integrated Memory Care (IMC) is a primary care practice for people living with dementia and their family caregivers. The practice integrates geriatric medicine, dementia specialty care/cognitive neurology, and palliative care along with caregiver services including support groups, classes, and psychotherapy for individuals and families. Finally, the practice hosts various opportunities to participate in caregiving research. Since 2015, the practice has treated over 2500 people living with dementia (PLWD) and supported their family caregivers. The mean age of patients is 78.6 years, the majority (66%) are female, and 42% identify as a racial or ethnic minoritized group. Caregiver relationship to patients (PLWD) is divided evenly between spouse (48%) and adult child (48%) and their mean age is 65.3 years. Men serving as primary family caregiver differ from their female counterparts in choice of clinical services, engagement in caregiver-specific programs, and participation in research. Successful marketing approaches include emphasis on caregiver as hero archetype, a clear message of the return-on-investment of services and activities. This presentation will describe the experience of supporting and engaging men as dementia family caregivers using aggregated data from multiple pilot studies as well as direct input from current active caregivers and IMC program staff.

